# Pathogenic spectrum and drug resistance of bloodstream infection in patients with acute myeloid leukaemia: a single centre retrospective study

**DOI:** 10.3389/fcimb.2024.1390053

**Published:** 2024-06-07

**Authors:** Han Wu, Manning Li, Chunyi Shou, Fangfang Shi, Xiaolu Song, Qingfeng Hu, Ying Wang, Yirui Chen, Xiangmin Tong

**Affiliations:** ^1^Graduate School of Clinical Medicine, Jinzhou Medical University, Jinzhou, Liaoning, China; ^2^Cancer Center, Department of Hematology, Zhejiang Provincial People’s Hospital, Affiliated People’s Hospital, Hangzhou Medical College, Hangzhou, Zhejiang, China; ^3^Department of Clinical Laboratory, Zhejiang Provincial People’s Hospital, Affiliated People’s Hospital, Hangzhou Medical College, Hangzhou, Zhejiang, China; ^4^Department of Central Laboratory, Affiliated Hangzhou First People’s Hospital, Xihu University, Hangzhou, Zhejiang, China; ^5^Department of Hematology, Affiliated Hangzhou First People’s Hospital, Xihu University, Hangzhou, Zhejiang, China

**Keywords:** acute myeloid leukaemia, bloodstream infections, distribution of pathogenic bacteria, drug resistance, prognosis, risk factors

## Abstract

**Background:**

Bloodstream infection (BSI) represent a prevalent complication in haematological malignancies (HMs). Typically, Patients with BSI usually undergo empirical treatment pending pathogen identification. The timely and effective management of BSIs significantly influences patient prognosis. However, pathogen distribution in BSIs exhibits regional variation. In this study, we investigated the clinical characteristics, pathogen spectrum, drug resistance, risk factors of short-term prognosis and long-term prognostic factors of acute myeloid leukemia (AML) patients with BSI at Zhejiang Provincal People’s Hospital.

**Methods:**

From 2019 to 2021, a total of 56 AML patients with BSI were treated in the Department of Haematology at Zhejiang Province People’s Hospital. Data regarding pathogen spectrum and drug resistance were collected for analysis. The patients were stratified into non-survivor cohort and survivor cohort within 30 days after BSI, and the predictors of 30-days mortality were identified through both univariate and multivariate Logistic regression analyses. Furthermore, Kaplan-Meier survival analysis and Cox regression analysis were employed to ascertain the risk factors associated with poor prognosis in AML patients complicated by BSI.

**Results:**

A total of 70 strains of pathogenic bacteria were isolated from 56 AML patients with BSI. Gram-negative bacteria constituted the predominant pathogens (71.4%), with *Klebsiella pneumoniae* being the most prevalent (22.9%). Gram-positive bacteria and fungi accounted for 22.9% and 5.7%, respectively. Univariate and multivariate analyses revealed significant differences in total protein, albumin levels, and the presence of septic shock between the non-survivor cohort and the survior cohort 30 days post-BSI. COX regression analysis showed that agranulocytosis duration exceeding 20 days (HR:3.854; 95% CI: 1.451–10.242) and septic shock (HR:3.788; 95% CI: 1.729–8.299) were independent risk factors for poor prognosis in AML patients complicated by BSI. Notably, the mortality rate within 30 days after *Stenotrophomonas maltophilia* infection was up to 71.4%.

**Conclusions:**

In this study, Gram-negative bacteria, predominantly Klebsiella pneumoniae, constituted the primary pathogens among AML patients with BSIs. Serum albumin levels and the presence of septic shock emerged as independent risk factors for mortality within 30 days among AML patients with BSI. In terms of long-term prognosis, extended agranulocytosis duration exceeding 20 days and septic shock were associated with elevated mortality rates in AML patients with BSI. Additionally, in our centre, *Stenotrophomonas maltophilia* infection was found to be associated with a poor prognosis. Early intervention for *Stenotrophomonas maltophilia* infection in our centre could potentially improve patient outcomes.

## Background

Acute myeloid leukaemia (AML) is a malignant proliferation of haematopoietic cells. The massive proliferation of abnormal blasts and immature cells in the bone marrow prevents normal haematopoiesis. Thus, the bone marrow and peripheral blood are characterised by an increase in immature leukocytes, mainly blasts (Assi et al., 2019; Wrighton, 2019). Immature cells accumulate in the bone marrow and replace normal bone marrow cells, megakaryocytes, and red blood cells, leading to loss of normal bone marrow function, bleeding, anaemia, and infection-related complications (McKenzie, 2005). Several factors related to the prognosis of patients with AML, such as infection, disease relapse, and haemorrhage. In addition, haematopoietic stem cell transplantation, chemotherapy regimens, and duration of agranulocytosis are closely related to patient prognosis. Among these, the incidence of bloodstream infections (BSIs) in haematological malignancies is 4–90% (Tumbarello et al., 2012; Samonis et al., 2013; Kolonen et al., 2017). Furthermore, the widespread utilisation of antibiotics has led to a gradual increase in the incidence of drug-resistant bacteria. Inappropriate empirical antimicrobial therapy or repeated infections contribute to the rise in the incidence of multiple drug resistance and mortality rates in patients (Moghnieh et al., 2015; Tang et al., 2020). Fortunately, by taking prompt preventive measures, we can significantly reduce the incidence of BSIs and lower the occurrence of related complications. This underscores the critical importance of early intervention. Such measures not only contribute to enhancing patient survival rates but also notably improve their overall quality of life, thereby creating a more conducive environment for their treatment. Hence, the significance of early BSIs prevention cannot be underestimated. However, the distribution of pathogenic bacteria in BSIs of patients in different regions varies greatly, which makes it difficult for early intervention of BSIs.

Gram-positive bacteria plays a predominant role as pathogens in certain haematology centres in Europe and America (Mikulska et al., 2014). However, the situation is markedly different in haematological wards in China, where Gram-negative bacteria are more prevalent (Deng et al., 2012). This disparity not only involves the types of pathogens but also extends to the establishment of medical infrastructure and the formulation of prevention and control strategies. Furthermore, regional variations also impact the antimicrobial susceptibility of specific pathogens (Deng et al., 2012; Mikulska et al., 2014). Resistance patterns of the same pathogenic bacteria may differ among different regions underscoring the need to consider the local microbial environment and resistance profiles when devising treatment plans and preventive strategies. This consideration is particularly crucial in the treatment of haematologic patients, where drug selection and administration must be tailored to the patient’s condition, the type of pathogenic bacteria, and the local susceptibility data. This integration ensures the optimal therapeutic outcomes and prognostic results (Deng et al., 2012; Mikulska et al., 2014). Therefore, as the etiological differences in different regions, there are some difficulties in empirical determination of medication for patients with BSIs and of prophylactic medication in patients at high risk of BSIs.

This study conducted a retrospective analysis of pathogen distribution, drug resistance, the risk factors associated with mortality within 30 days of BSI, and long-term prognosis of AML patients with BSI in a subset of 56 AML patients with BSIs, including 18 cases that resulted in death. This study provides a robust basis for clinically determining treatment approaches for AML patients with concurrent BSI and for improving patient prognosis in China.

## Materials and methods

### Patient characteristics

A retrospective analysis was conducted on 56 AML patients who received treatment at the Hematology Department of Zhejiang People’s Hospital. The deadline for follow-up is January 1, 2024. The age, sex, platelet count, activated partial thromboplastin time, total protein, albumin, glucocorticoid, septic shock, agranulocytosis duration, agranulocytosis to infection, history of infection, prior HSCT history and chemotherapy cycles were collected from 56 patients. Patients who died within 30 days after BSI constituted non-survivor cohort, while those who still survive formed the survivor cohort. Patients in the cohort who died within 30 days after BSI had an average age of 48 years, with 9 males and 9 females. In the other cohort, the average age was 52 years, with 25 males and 18 females. (**Table 1**).

**Table 1:** Laboratory parameters and baseline of 56 AML patients with BSI.

Variables	Total (n=61)	Survivors (n=43)	Non-survivors (n=18)	P
Age (years)	51 (45–59)	52 (45–59.5)	48 (42.5–55.25)	0.374
Sex				0.585
Male	34 (55.7)	25 (58.1)	9 (50)	
Female	27 (44.3)	18 (41.9)	9 (50)	
Neutrophils (10^9/L)	0.01 (0–0.1)	0.01 (0–0.1075)	0.01 (0–0.0775)	0.91
Plt (10^9/L)	10 (4.5–25.5)	13 (5–27.5)	7.5 (3–18)	0.051
APTT (s)	30.7 (27.7–36.4)	29.1 (27.6–34.1)	36.8 (32.55–45.075)	0.003
Dbil (umol/L)	5.0 (2.7–8.4)	4.15 (2.7–6.2)	8.4 (4.1–13.15)	0.016
Total protein (g/L)	54.99 ± 8.52	57.695 ± 7.385	48.682 ± 8.061	<0.01
Albumin (g/L)	31.264 ± 5.632	33.295 ± 4.653	26.594 ± 5.075	<0.01
Glucocorticoid				0.781
Yes	35 (57.3)	24 (55.8)	11 (61.1)	
No	26 (42.7)	19 (44.2)	7 (38.9)	
Septic shock				0.018
Yes	22 (36.1)	11 (25.6)	11 (61.1)	
No	39 (63.9)	32 (74.4)	7 (38.9)	
Agranulocytosis duration				0.002
> 20 days	32 (52.5)	17 (39.5)	15 (83.3)	
≤ 20 days	29 (47.5)	26 (60.5)	3 (16.7)	
Agranulocytosis to infection				0.140
> 2 weeks	21 (34.4)	12 (27.9)	9 (50)	
≤ 2 weeks	40 (65.6)	31 (72.1)	9 (50)	
History of infection				0.781
Yes	46 (75.4)	32 (74.4)	14 (77.8)	
No	15 (24.6)	11 (25.6)	4 (22.2)	
Prior HSCT				0.572
Yes	26 (42.6)	17 (39.4)	9 (50)	
No	35 (56.4)	26 (60.5)	9 (50)	
Chemotherapy cycles				0.575
>5	29 (47.5)	19 (44.2)	10 (55.6)	
≤ 5	32 (52.5)	24 (55.8)	8 (44.4)	

Plt, Platelet; APTT, Activated partial thromboplastin time; Dbil, Direct bilirubin; HSCT, haematopoietic stem cell transplantation.

In addition, the pathogens of AML patients with BSI in the Hematology Department of Zhejiang people’s Hospital from 2013 to 2021 and the mortality within 30 days after bloodstream infection were collected retrospectively.

### Diagnostic criteria and definitions

#### Diagnostic criteria for BSIs

1. Body temperature > 38.0°C or < 36.0°C, possibly accompanied by chills; aerobic bacteria, anaerobic bacteria, and fungal culture should be completed before the use of antibacterial drugs or during high fever and chills.
2. Poisoning symptoms without obvious infection lesions: Body temperature > 38.0°C or < 36.0°C may be accompanied by chills hypotension, oliguria or high lactic acid blood levels, but no pathogens were isolated from respiratory tract, urinary tract, gastrointestinal tract and skin.
3. Antigen substance of pathogenic bacteria detected in blood culture is consistent with signs and symptoms: Pathogenic microorganisms were isolated from blood culture, accompanied by one of the following symptoms: fever, chills, hypoxia and hypotension (China MoHotPsRo, 2005).
4. A*granulocytosis*: absolute neutrophil count (ANC) in peripheral blood < 0.5×10^9^/L or ANC < 0.5×10^9^/L predicted to occur after 48 hours (Chen Xin, 2020).

#### The diagnostic criteria of AML

Peripheral blood or bone marrow blasts ≥ 20% or peripheral blood or bone marrow blasts < 20%, with t (8, 21) (q22;q22), inv (16) (p13q22), t (16; 16) (p13;q22), t (15; 17) (q22;q12) or other recurrent genetic abnormalities (Leonard et al., 2017).

#### The diagnostic criteria of *septic shock*

Septic shock exhibits low blood pressure necessitating vasopressor therapy to maintain an average blood pressure of 65 mmHg or higher, or serum lactate levels are greater than 2 mmol/L after full fluid resuscitation (Shankar-Hari et al., 2016).

### Inclusion and exclusion criteria

#### Inclusion criteria

1. Patient diagnosed with AML.
2. Positive blood culture results.

#### Exclusion criteria

1. Patient diagnosed with non-AML.
2. Patients who fail to perform blood culture or whose blood culture results are negative.
3. Patients with multiple positive blood cultures with the same pathogen during the same hospitalisation were recorded as once.

### Statistical methods

All variables were statistically processed using SPSS software (version 25.0). Non-normal distribution was expressed as median and interquartile range, while normal distribution was expressed as mean ± standard deviation *P* < 0.05 was considered statistically significant. The distribution of pathogen spectrum between groups was analysed by Fisher’s exact test. Independent sample T-tests and logistic regression analysis were applied to assess the risk factors impacting the prognosis within 30 days after infection. Additionally, Kaplan-Meier survival analysis and Cox regression analysis were conducted to identify risk factors for poor prognosis of AML patients with BSI.

## Results

### Distribution of pathogenic microorganisms

66 strains of bacteria and 4 strains of fungi were isolated from 70 strains, of which 50 were Gram-negative bacteria (71.4%). Specifically, there were 16 strains of *Klebsiella pneumoniae* (22.9%), 10 strains of *Escherichia coli* (14.2%), 9 strains of *Pseudomonas aeruginosa* (12.9%), 7 strains of *Stenotrophomonas maltophilia* (10%), 2 strains of *Enterobacter cloacae* (2.9%), 1 strain each of *Proteus vulgaris*, *Enterobacter aerogenes, Aeromonas sobria*, *Oral ciliates*, *Klebsiella oxytoca* and *Meningeal Septicemia Elisabia* (1.4%, respectively). Additionally, there are 16 strains of Gram-positive bacteria (22.9%), 5 strains were *Enterococcus faecium* (7.1%), and 2 strains each of *Streptococcus mitis*, *Staphylococcus epidermidis*, *Staphylococcus hominis subsp hominis*, and *Bacillus cereus* (2.9%), and 1 strain each of *Staphylococcus aureus*, *Hemolytic Staphylococcus*, and *Corynebacterium Gerschi*(1.4%). 4 strains of fungi were *Candida tropicalis* (5.7%) (**Table 2**).

**Table 2:** Distribution of pathogenic bacteria (70 strains) in 56 patients with acute myeloid leukaemia with BSI.

Pathogenic bacteria	Number	Rate (%)
Gram-negative	50	71.4
*Klebsiella pneumoniae*	16	22.9
*Escherichia. coli*	10	14.2
*Pseudomonas aeruginosa*	9	12.9
*Stenotrophomonas maltophilia*	7	10
*Enterobacter cloacae*	2	2.9
*Proteus vulgaris*	1	1.4
*Enterobacter aerogenes*	1	1.4
*Aeromonas sobria*	1	1.4
*Oral ciliates*	1	1.4
*Klebsiella oxytoca*	1	1.4
*Meningeal Septicemia Elisabia*	1	1.4
Gram-positive bacteria	16	22.9
*Enterococcus faecium*	5	7.1
*Streptococcus mitis*	2	2.9
*Staphylococcus epidermidis*	2	2.9
*Staphylococcus hominis subsp hominis*	2	2.9
*Bacillus cereus*	2	2.9
*Staphylococcus aureus*	1	1.4
*Hemolytic Staphylococcus*	1	1.4
*Corynebacterium Gerschi*	1	1.4
Fungus	4	5.7
*Candida Tropicalis*	4	5.7

Compared to the period of 2013–2015, where there were no reported cases, the incidence of Carbapenem-Resistant Enterobacter (CRE) has shown a steady increase, reaching 5% during 2016–2018 and escalating to 11.4% during 2019–2021. Similarly, the incidence of Extended-Spectrum β-Lactamases (ESBL) (22.5%) and Multi-Drug Resistance (MDR) (15%) during 2016–2018 surpassed that of ESBL (14.9%) and MDR (0%) during 2013–2015, as well as exceeding the rates of ESBL (7.1%) and MDR (11.4%) during 2019–2021. However, the mortality rate within 30 days after MDR infection during 2019–2021 (25%) was higher than that observed in 2013–2015 (0%) and 2016–2018 (16.7%) (**Supplementary Table 1**).

### Pathogen drug resistance

Gram-negative bacteria: There were 16 strains of *Klebsiella pneumoniae*, with a resistance rate of over 65% to cephalosporins, quinolones, and penicillins. *Escherichia coli* is sensitive to tigecycline, and the drug resistance rate is 0%. The resistance rate to piperacillin/tazobactam, ceftazidime/sulbactam, ceftolozane/tazobactam, ertapenem, and amikacin was 10%. It exhibits a resistance rate of 60% to ampicillin/Sulbactam, ciprofloxacin, levofloxacin, sulfamethoxazole/trimethoprim (TMP-SMX), and the resistance rate to ampicillin was as high as 80%. The resistance rate of *Pseudomonas aeruginosa* to piperacillin/tazobactam, ceftazidime, cefepime, cefoperazone/sulbactam, meropenem, ciprofloxacin, and levofloxacin was 55.6% (**Table 3**). Eight strains of CRE exhibited complete resistance to piperacillin/tazobactam, ceftriaxone, cefepime, cefoperazone, ciprofloxacin, and levofloxacin but showed sensitivity to amikacin. (**Supplementary Table 2**). Six strains of CRE were detected from sixteen strains of *Klebsiella pneumoniae*. Ten strains of *Escherichia coli* produced three strains of ESBL. Five of nine *Pseudomonas aeruginosa* strains produced carbapenem-resistant Pseudomonas aeruginosa (CRPA). There were eight strains of MDR, including two strains of *Klebsiella pneumoniae*, three strains of *Escherichia coli*, two strains of *Enterococcus faecium* and one strain of *Streptococcus mitis* (**Supplementary Table 3**).

**Table 3:** Resistance rate of major Gram-negative bacilli to antimicrobial agents (%).

Antibacterial drugs	*Klebsiella pneumoniae*	*Escherichia coli*	*Pseudomonas aeruginosa*
Ampicillin	–	8(80)	–
Ampicillin/Sulbactam	–	6(60)	–
Piperacillin/Tazobactam	11(68.8)	1(10)	5(55.6)
Cefazolin	13(81.2)	5(50)	–
Ceftazidime	–	3(30)	5(55.6)
Ceftriaxone	12(75)	4(40)	–
Cefepime	12(75)	3(30)	5(55.6)
Cefotetan	–	1(10)	–
Cefoperazone	12(75)	1(10)	5(55.6)
Aztreonam	12(75)	3(30)	–
Ertapenem	–	1(10)	–
Meropenem	8(50)	1(10)	5(55.6)
Amikacin	4(25)	1(10)	–
Gentamicin	7(43.8)	3(30)	0
Tobramycin	7(43.8)	2(20)	0
Ciprofloxacin	13(81.2)	6(60)	5(55.6)
Levofloxacin	11(68.8)	6(60)	5(55.6)
TMP-SMX	10(62.5)	6(60)	–
Tigecycline	–	0(0)	–

TMP-SMX, Sulfamethoxazole/Trimethoprim; “-”, without drug sensitivity.

Gram-positive bacteria: *Enterococcus faecium* and *Staphylococcus haemolyticus* aureus demonstrate a resistance rate of 0% to linezolid, vancomycin, daptomycin, and tigecycline. They exhibit a 100% resistance rate to penicillin. The five strains of *Enterococcus faecium* were resistant to ampicillin and penicillin and had high resistance to erythromycin and high concentration of gentamicin (**Supplementary Table 4**).

Fungi: A total of four strains of fungi were isolated, all of which were *Candida tropicalis*, and drug sensitivity test was not performed.

### Outcome

In 56 patients with AML, with 18 deaths within 30 days after BSI. Among these 18 patients, 5 cases were infected by *Pseudomonas aeruginosa*, 4 cases by *Klebsiella pneumoniae*, 3 cases by *Escherichia coli*, 1 case by *Candida tropicalis*, 1 case by *Enterobacter cloacae*, 1 case by *Staphylococcus aureus*, 1 case by *Enterococcus faecalis*, and 1 case by *Staphylococcus epidermidis*.

### Risk factors for death within 30 days after AML complicated with BSI

Univariate analysis showed that, compared to the non-survivor cohort, survior cohort had lower activated partial thromboplastin time and direct bilirubin levels. Additionally, total protein and albumin levels, the number of patients without septic shock, and the number of patients with neutrophil reduction lasting more than 20 days were all higher than those in the non-survivor cohort (P<0.05) (**Table 1**). Following logistic regression, significant differences were observed between the two cohorts in terms of total protein (P=0.017, HR: 0.861; 95% CI: 0.761–0.974), albumin (P=0.005, HR: 0.740; 95% CI: 0.599–0.914), and septic shock (P=0.046, HR: 4.399; 95% CI:1.025–18.870) (**Table 4**).

**Table 4:** Multivariate Analysis of Risk Factors for poor outcome within 30 days after infection.

Variables	Multivariate analysis	
	OR (95%CI)	*P*
APTT	1.096 (0.969–1.240)	0.145
Dbil	0.996 (0.935–1.062)	0.910
Total protein	0.861 (0.761–0.974)	0.017
Albumin	0.740 (0.599–0.914)	0.005
Septic shock	4.399 (1.025–18.870)	0.046
Agranulocytosis duration	2.102 (0.392–11.278)	0.386

APTT, Activated partial thromboplastin time; Dbil, Direct bilirubin.

### Survival analysis of AML complicated with BSI

**Figure 1:**
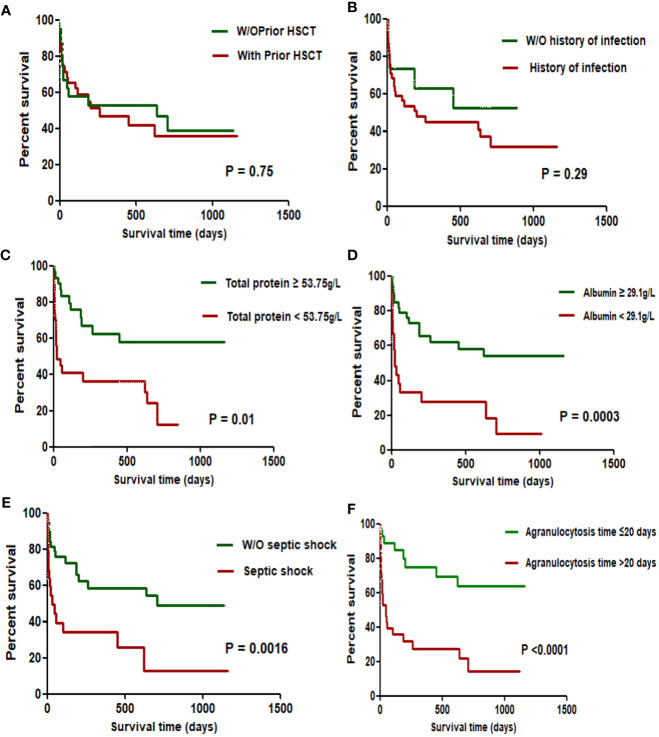
Survival Analysis of AML Complicated with BSI. Each label has the following meanings:the Impact of Prior Hematopoietic stem cell transplantation **(A)**, History of infection **(B)**, Total protein **(C)**, Albumin **(D)**, Septic shock **(E)** and Agranulocytosis time **(F)** on the Prognosis of AML Patients with BSI. W/O: without; HSCT: Hematopoietic stem cell transplantation.

In addition to logistic regression analysis, factors that were clinically considered to be related to the long-term prognosis of patients with infection were added for survival analysis. Kaplan-Meier survival analysis showed that prior HSCT history and history of infection were not related to the poor prognosis of AML patients with BSI (**Figures 1A, B**). Total protein, albumin, septic shock and agranulocytosis duration>20 days were related to the poor prognosis of AML patients after BSI (**Figures 1C–F**). COX regression analysis showed that agranulocytosis duration >20 days (HR:3.854; 95% CI: 1.451–10.242) and septic shock (HR:3.788; 95% CI: 3.1.729–8.299) were predictors of poor long-term prognosis in patients with AML after BSI (**Table 5**).

**Table 5:** Multivariate Cox regression analysis of AML Complicated with BSIs.

Variables	Multivariate analysis	
	OR (95%CI)	*P*
Total protein	0.711 (0.25–2.02)	0.523
Albumin	0.629 (0.235–1.685)	0.356
Septic shock	3.788 (1.729–8.299)	0.001
Agranulocytosis duration	3.854 (1.451–10.242)	0.007

## Discussion

BSI is a serious systemic infectious disease caused by various pathogenic microorganisms that invade the blood circulation and release toxins and metabolites (Jang-yong et al., 2020). It is one of the common complications of AML patients and is associated with poor prognosis (Collin et al., 2001; Nørgaard et al., 2006). Leukopenia, especially the decrease in neutrophils, can be caused by patients with AML themselves and chemotherapy, which leads to immunodeficiency, and the incidence of BSIs is significantly increased. Previous exposure to antibiotics or inappropriate empirical antibiotic treatment contributes to an increase in the incidence of BSI, drug resistance and mortality. This trend was corroborated by Italian studies conducted by Gudiol C et al. and Trecarichi EM et al., wherein the incidence of MDR escalated from 13.7% in 2011 to 31.5% in 2023, accompanied by an increase in mortality rates from 24% to 34.4% (Gudiol et al., 2011; Trecarichi et al., 2023).

The distribution of types and drug resistance rates of pathogenic bacteria of BSIs varies in different regions and times. Studies have shown that Gram-positive bacteria were the most common cause of BSIs in cancer patients before 2000, and the infection rate of Gram-positive bacteria reached 76% in 2000 (Wisplinghoff et al., 2003; Ortega et al., 2005). Trecarichi et al. found that the pathogenic bacteria of BSIs in patients with HMs changed from Gram-positive bacteria to Gram-negative bacteria from 2006 to 2016 (Trecarichi and Tumbarello, 2014; Trecarichi et al., 2015). A study on BSIs in 357 AML patients in Finland from 2003 to 2011 showed that most of the pathogens were Gram-positive bacteria (65.7%), with coagulase-negative staphylococci accounting for the highest proportion at 24.7% among Gram-positive bacteria (Kolonen et al., 2017). An Italian study on adult haematological malignancies from 2009 to 2012 revealed that Gram-negative bacteria constituted 52.8% of infections (Trecarichi et al., 2015).

Gram-negative bacteria have been the main cause of infection in related studies in China, of which *Escherichia coli* is the most common (Chen et al., 2010; Li et al., 2022). *Escherichia coli* typically colonizes in the gastrointestinal tract. During the treatment of AML, cytotoxic chemotherapy alters the integrity of the gastrointestinal mucosa and impairs the immune system, increasing the risk of invasive infections caused by the translocation of colonising bacteria (Montassier et al., 2015; Garcia-Vidal et al., 2018). Similarly, gram-negative bacteria are the main pathogens in our centre. However, in our study, *Klebsiella pneumoniae* took precedence among Gram-negative bacteria. Some patients with potential prior infection, having received β-lactam cephalosporins and quinolones antibiotics for infection treatment, exhibited *Klebsiella pneumoniae* in the screening. Previous antimicrobial therapy, particularly β-lactams and cephalosporins, can also elevate the risk of multidrug resistance, leading to a high incidence of *Klebsiella pneumoniae* in the region (Garcia-Vidal et al., 2018). This emphasizes the need for cautious use of broad-spectrum antibiotics in our centre. In the study by Chen et al., the results are similar to those of this study, with *Klebsiella pneumoniae* being the predominant gram-negative bacterium (Esbenshade et al., 2015; Chen et al., 2019). In our centre, the predominant pathogen spectrum comprises Gram-negative bacteria. Standard empirical treatment typically involves broad-spectrum β-lactam antibiotics, while *enterococci* exhibit complete resistance to β-lactam antibiotics (Kristich et al., 2014; Gagetti et al., 2019). Consequently, the incidence of *enterococci* has exhibited a progressive increase from 2013 to 2021 in our centre. *Enterococci* has become predominant Gram-positive bacteria from 2019 to 2021 in our centre, likely attributed to the inadequacy of empirical antimicrobial therapy to address *enterococcal* infections. In cases where a Gram-positive bacterial infection is suspected, the addition of vancomycin to empirical treatment regimens may be considered.

In other studies, Gram-positive bacteria are mainly represented by *Streptococcus viridans* and *Staphylococcus epidermidis* (Chen et al., 2019; Carvalho et al., 2020), which is totally different from the situation in our centre. The clinical medication principle involves tailoring treatments based on blood culture and susceptibility results to ensure efficacy while minimising the development of drug-resistant pathogens. However, blood pathogen cultures and susceptibility testing can be time-consuming. Clinical treatment for BSI often relies on empirical approaches. As antimicrobial drug usage increases, pathogen resistance grows, making empirical anti-infection treatment more challenging. Thus, understanding the microbial antimicrobial spectra and resistance profiles in different healthcare centres is crucial for guiding clinical empirical drug administration, improving susceptibility testing, and reducing overall risk.

In our centre, the resistance rate of *Klebsiella pneumoniae* to cephalosporins and quinolones was high, and the resistance rate to aminoglycosides was low. The resistance rates of *Escherichia coli* to β-lactam/β-lactamase inhibitors, sulfonamides and cephalosporins were high, their resistance rate to carbapenems was low.

Therefore, aminoglycosides and carbapenems can be recommended for AML patients with BSI suspected of being infected with Gram-negative bacteria, which consistent with the findings of Trecarichi et al (Trecarichi et al., 2015; Åttman et al., 2021). Although the predominance of Gram-positive bacteria in our study differs from other studies, consistent with our results, most of them exhibited high sensitivity to vancomycin (Chen et al., 2019; Li et al., 2022). Therefore, in our centre, empirical treatment with vancomycin may be considered for AML patients suspected of Gram-positive bacterial infection prior to obtaining definitive pathogen and drug sensitivity results.

Studies conducted by Gudiol C et al. have highlighted the consequences of the inappropriate use of antibiotics, demonstrating an associated increase in drug resistance (Gudiol et al., 2011; Trecarichi et al., 2023). Comparing drug resistance rates from 2013 to 2021 in our centre revealed a gradual upward trend, attributed to the use of carbapenem in empirical anti-infective therapy. Specifically, the incidence of ESBL and MDR increased during the period of 2016–2018 compared to 2013–2015, potentially linked to the irrational use of antibiotics. Kollef MH et al. found that prolonged antibiotic exposure was a risk factor for MDR (Munoz-Price et al., 2016; Kollef et al., 2021). Interestingly, our study observed a decreasing trend in MDR incidence during 2019–2021 compared to 2016–2018, potentially attributed to reduce antibiotic exposure duration and decreased use of empirical antibacterial drugs in our centre.

Frequent exposure to initially inappropriate empirical antibiotic treatment may play a pivotal role in the risk of MDR-related death, hospital bacterial colonisation or infection and viral reactivation are also closely related to MDR morbidity and mortality (Hotchkiss et al., 2013; Zilberberg et al., 2014; van Vught et al., 2016). Compared with non-MDR infection, patients with MDR bacterial infection have an increased risk of mortality (Cornaglia et al., 2011; Vardakas et al., 2013). Our data also revealed an increased mortality rate within 30 days attributable to MDR infections, despite a declining trend in MDR incidence. However, due to the limited sample size, further data collection is warranted for comprehensive research. Among the 8 patients with MDR infection from 2019 to 2021, only one patient had previous colonisation of MDR strain (*Enterobacter asburiae*), which differed from the MDR strain (*Escherichia coli*) causing BSI. This patient received antifungal treatment combined with cephalosporin, with subsequent adjustment of antibiotic therapy based on drug sensitivity results. Prior to the identification of the causative pathogen in bloodstream infections, 6 out of the remaining 7 patients received empirical antifungal therapy, primarily in combination with β-lactam, carbapenem, or quinolone antibiotics, until the pathogens of BSI were identified.

Septic shock, denotes a complex syndrome of septicemia induced by microbial agents and their toxic byproducts, resulting in shock (Shankar-Hari et al., 2016). The use of chemotherapeutic drugs leads to neutropenia, which leads to severe immunodeficiency and increases the probability of septic shock (Na et al., 2022).The incidence of septic shock in patients with HMs ranged from 20.7% to 31% (Royo-Cebrecos et al., 2022; Wang et al., 2023; Zhang et al., 2023). In this study, the incidence of septic shock was 37.3%, with 50% of cases resulting in death within 30 days after BSI. The elevated incidence of septic shock in our centre may be associated with neutropenia occurrence among patients, with over half of them experiencing neutropenia lasting over 20 days (Na et al., 2022; Islas-Muñoz et al., 2024).

In addition, coagulation disorders associated with haematological malignancies can also increase mortality during septic shock (Lemiale et al., 2023). This study found that septic shock or the use of vasoactive drugs is an independent risk factor for the death of patients with AML after BSI. Royo-Cebrecs et al’ s study is similar to our findings (Chen et al., 2020; Royo-Cebrecos et al., 2022).

Albumin plays a crucial role in mitigating inflammation, maintaining vascular endothelial integrity, and regulating acid-base balance. It also acts to inhibit the progression of inflammation and damage to microcirculation and tissues (Ferrer et al., 2018; Hariri et al., 2018). In the context of infection, capillary permeability increases, causing albumin to seep from the plasma into the interstitial space, resulting in hypoalbuminemia (Gradel et al., 2018). In this study, lower levels of albumin were found to be indicative of adverse outcomes within 30 days following BSI. Notably, research conducted by Benjamin M. Greenberg and others has demonstrated a linear association between albumin levels and the prognosis of bacteremic patients (Greenberg et al., 2005; Magnussen et al., 2016). Diminished albumin levels in patients can lead to immunosuppression following BSI, contributing to poorer patient outcomes (McSorley et al., 2017). Furthermore, low albumin levels in patients with potential infectious foci can lead to infection recurrence (Leal et al., 2018; Abdel-Razik et al., 2020). At the same time, reduced albumin levels are associated with a decreased effectiveness of antibiotic treatment (Ibrahim et al., 2000; Holmes et al., 2018). Low albumin levels serve as a risk factor for treatment failure in Gram-negative bacteremia (Hashiguchi et al., 2020).

Agranulocytosis refers to neutropenia, bone marrow suppression caused by the use of chemotherapeutic drugs or by haematological tumours hinders the generation of an effective immune response, and patients with reduced immunity are prone to infection. The longer the agranulocytosis, the higher the incidence of infection, and the mortality rate of blood stream infection in patients with agranulocytosis and fever in patients with HMs can reach 42% (Zuber et al., 2012; Wohlfarth et al., 2014). The longer the duration of agranulocytosis, the worse the prognosis of HMs patients with infection (Darmon et al., 2002; Li et al., 2018). This study also confirmed this result. However, this study suggests that the duration of neutropenia is not an independent risk factor for death within 30 days after BSI. Tang’s study is consistent with our findings (Tang et al., 2018).This may be due to the early application of empirical antibacterial drugs to timely control the infection caused by agranulocytosis, which reduced the incidence of serious complications and mortality (Falcone et al., 2020; Zhang et al., 2020). This correlation requires additional data for further study and verification.

*Stenotrophomonas maltophilia* is an opportunistic pathogen that often occurs in hospitals and in immunosuppressed patients (Looney et al., 2009; Adegoke et al., 2017). The infection rate of this bacterium increased gradually because of the low immune function and the wide use of broad-spectrum antibiotics (Said et al., 2024). TMP-SMX is the first choice of treatment for *Stenotrophomonas maltophilia* infection, but TMP-SMX treatment may cause myelosuppression, hyperkalaemia and hypersensitivity syndrome, and *Stenotrophomonas maltophilia* infection is relatively rare. Hence, TMP-SMX is not generally used for empirical treatment of BSIs (Brooke, 2012; Chang et al., 2015). In this study, seven patients with *Stenotrophomonas maltophilia* infection were included, and no drug sensitivity test was performed. Five patients died within 30 days after BSI with *Stenotrophomonas maltophilia*, with a mortality rate of 71.4%. Sumida et al. also reported similar findings that *Stenotrophomonas maltophilia* infection was associated with adverse outcomes in patients (Cho et al., 2014; Sumida et al., 2015). A history of recurrent infections and prior antibiotic use are risk factors for *Stenotrophomonas maltophilia* infection (Apisarnthanarak et al., 2003; Bao et al., 2020). In our study, all the patients with *Stenotrophomonas maltophilia* infection had a history of repeated infection before BSI, but no multiple infection between *Stenotrophomonas maltophilia* and other bacteria were found during BSI (Apisarnthanarak et al., 2003; Bao et al., 2020). Among seven patients with *Stenotrophomonas maltophilia* infection, 5 patients who died within 30 days after BSI had a history of empirical use of carbapenem antibiotics before the pathogen was identified. *Stenotrophomonas maltophilia* exhibits resistance to some empirically used clinical treatments, particularly β-lactam antibiotics (including carbapenems) and aminoglycosides. As a result, routine empiric therapy often fails to achieve timely and effective control of the *Stenotrophomonas maltophilia* infection before blood culture results become available (Gales et al., 2019; Mojica et al., 2019).

Nonetheless, these results must be interpreted with caution and a number of limitations should be borne in mind. This study is a single-centre study in Zhejiang Province People’s Hospital, with limited sample size and certain regional characteristics. Thus, patients from other places were still need to be included. In addition, some factors that have been associated with the prognosis of patients with post-infection AML in other studies were not relevant in this study and may require confirmation with a larger sample size.

## Conclusion

In this study, 56 AML patients complicated by BSIs were primarily infected with Gram-negative bacteria. Aminoglycosides and carbapenems are recommended for empirical treatment based on the pathogenic spectrum and drug resistance status, pending definitive drug sensitivity results. Serum albumin levels and the presence of septic shock emerged as independent risk factors for mortality within 30 days among AML patients with BSI. Furthermore, prolonged agranulocytosis exceeding 20 days and septic shock were associated with heightened mortality rates in terms of long-term prognosis for AML patients with BSI. In addition, *Stenotrophomonas maltophilia* infection may be associated with poor prognosis, with TMP-SMX serving as a potential treatment option to manage infection and enhance patient outcomes.

## Data availability statement

The raw data supporting the conclusions of this article will be made available by the authors, without undue reservation.

## Ethics statement

The study was approved by the research involving humans were approved by the ethics committee of Zhejiang people's Hospital (QT2023130). The studies were conducted in accordance with the local legislation and institutional requirements. Written informed consent for participation was not required from the participants or the participants’ legal guardians/ next of kin in accordance with the national legislation and institutional requirements.

## Author contributions

HW: Software, Validation, Writing – original draft, Writing – review & editing. ML: Writing – original draft, Data curation, Writing – review & editing. CS: Data curation, Writing – review & editing, Formal analysis. FS: Writing – review & editing, Data curation. XS: Writing – review & editing. QH: Data curation, Writing – review & editing. YW: Writing – review & editing. YC: Writing – review & editing. XT: Writing – review & editing.
