# Supplementary material for: Pathogenic spectrum and drug resistance of bloodstream infection in patients with acute myeloid leukaemia: a single centre retrospective study
Source: Front Cell Infect Microbiol. 2024 Jun 7;14:1390053. doi: 10.3389/fcimb.2024.1390053 (PMC11190328; doi:10.3389/fcimb.2024.1390053)
Supplement: Supplementary file 1 [file Table_1.docx]

**Table 1** Incidence rate of CRE, ESBL and MDR in 2013-2021(%)

|  | CRE | ESBL | MDR | Mortality within 30 days after MDR infection |
| --- | --- | --- | --- | --- |
| 2013-2015 | 0 | 14.9 | 0 | 0 |
| 2016-2018 | 5 | 22.5 | 15 | 16..7 |
| 2019-2021 | 11.4 | 7.1 | 11.4 | 25 |

CRE: Carbapenem-resistant Enterobacter; ESBL: Extended-Spectrum β-Lactamases; MDR: Multi-drug resistant

**Table 2** Susceptibility and resistance of 8 strain of CRE (carbapenem-resistant Enterobacter) to antimicrobial agents (%)

| **Antibacterial drugs** | **Sensitivity rate** | **Drug resistance rate** |
| --- | --- | --- |
| Piperacillin/Tazobactam | 0（0） | 8（100） |
| Ceftriaxone | 0（0） | 8（100） |
| Cefepime | 0（0） | 8（100） |
| Cefoperazone | 0（0） | 8（100） |
| Ciprofloxacin | 0（0） | 8（100） |
| Levofloxacin | 0（0） | 8（100） |
| Aztreonam | 1（0） | 7（100） |
| Meropenem | 1（12.5） | 7（87.5） |
| Amikacin | 7（87.5） | 1（12..5） |
| Gentamicin | 3（37.5） | 5（62.5） |
| TMP-SMX | 3（37.5） | 5（62.5） |

TMP-SMX: Sulfamethoxazole/Trimethoprim

**Table 3** Distribution of CRE, ESBL, CRPA and MDR in Gram-negative bacteria

|  | CRE（n=8） | ESBL（n=5） | CRPA（n=5） | MDR（n=8） |
| --- | --- | --- | --- | --- |
| *Klebsiella pneumoniae* | 6 | 2 | 0 | 2 |
| *Escherichia. coli* | 1 | 3 | 0 | 3 |
| *Pseudomonas aeruginosa* | 0 | 0 | 5 | 0 |
| *Enterobacter cloacae* | 1 | 0 | 0 | 0 |
| *Enterococcus faecium* | 0 | 0 | 0 | 2 |
| *Streptococcus mitis* | 0 | 0 | 0 | 1 |

CRE: Carbapenem-resistant Enterobacter; ESBL: Extended-Spectrum β-Lactamases; CRPA: Carbapenem-resistant Pseudomonas aeruginosa; MDR: Multi-drug resistant

**Table 4** Antibiotic resistance of *Enterococcus faecium* (%)

| **Antibacterial drugs** | **Drug resistance rate** |
| --- | --- |
| Ampicillin | 100 |
| Penicillin | 100 |
| Erythromycin | 80 |
| Linezolid | 0 |
| Vancomycin | 0 |
| Pudim / Daffodil | 0 |
| Tigecycline | 0 |
| High concentration gentamicin | 60 |
| High concentrations of streptomycin | 20 |
